# Dynamic hip screws versus cannulated screws for femoral neck fractures: a systematic review and meta-analysis

**DOI:** 10.1186/s13018-020-01842-z

**Published:** 2020-08-26

**Authors:** Lang Li, Xue Zhao, Xiaodong Yang, Xueyang Tang, Ming Liu

**Affiliations:** 1 Department of Pediatric Surgery, West China Hospital, Sichuan University, Chengdu, 610041 Sichuan People’s Republic of China; 2 Department of Obstetrics and Gynaecology, The First Affiliated Hospital of Chongqing Medical University, Chongqing, 400016 People’s Republic of China; 3 Department of Orthopedics, West China Hospital, Sichuan University, #37, Guo Xue Xiang, Chengdu, 610041 Sichuan People’s Republic of China

**Keywords:** Meta-analysis, Dynamic hip screw, Cannulated screws, Femoral neck fractures

## Abstract

**Objective:**

Dynamic hip screw (DHS) and cannulated screws (CS) are widely used for femoral neck fractures. However, there is no definite result as to which surgical method bring less complications. We performed this study to compare the complication (mortality, non-union, avascular necrosis (AVN), and revision) of DHS and CS for the treatment of femoral neck fractures patients.

**Methods:**

We searched Pubmed, Ovid, Cochrane Central Register of Controlled Trials, and other relevant studies related the comparison of DHS versus CS for femoral neck fractures from inception to Jan 7, 2020. The quality of the included randomized controlled trials (RCTs) and retrospective studies were assessed using the Cochrane Collaboration tool and Newcastle-Ottawa (NOS), respectively. The meta-analysis was performed by the RevMan 5.2 software.

**Results:**

Nine RCTs and seven retrospective cohort studies were included for meta-analysis. CS was found to be superior to DHS with respect to AVN rate (OR 1.47; 95% CI 1.08–1.99; *p* = 0.01, *I*^*2*^ = 0%). There were no significant between-group differences with respect to mortality, non-union, and revision (*p* > 0.05).

**Conclusion:**

DHS and CS have similar complication including mortality, revision rate, and non-union, but CS has superior to DHS on ANV. However, further studies are required to provide more robust evidence owing to some limitations.

## Introduction

Femoral neck fractures are common fractures in the orthopedics department, and incidence of femoral neck fracture increased with the increase of population ages and traffic accidents. Previous study reported that for young adult patients, the incidence of femoral neck fractures amounted to 0.04%. However, the incidence for older patients is increase to 0.28~0.64% [1, 2]. In the past, due to the limited treatment methods for femoral neck fracture, it has been considered as “unresolved fracture” [3]. Previous studies reported that femoral neck fractures are associated with complications such as avascular necrosis (AVN), non-union, implant failure/revision, and even death [4, 5].

There are many options to treat femoral neck fracture. Previous studies reported that femoral neck fractures with following surgery are associated implant failure [6, 7]. Multiple cannulated screws (CS) and dynamic hip screw (DHS) are widely used for non-displaced or young patients. CS has better biomedical properties such as anti-rotation and less invasive, which was widely used in non-displaced intracapsular fractures [6, 8]. DHS could maintain the neck-shaft angle and anatomical reduction, which is helpful for fracture fixation [7]. Lee et al. reported that DHS has a higher rate of overall success when compared to the MCS group [9]. However, a recent study showed no significant difference between two treatments on rates of revision surgery and complications [10]. In addition, systematic review or meta-analysis is also conducted in previous studies [8, 11]. However, whether CS is superior to DHS was not consistent. Moreover, some limitations exist in previous studies: the sample size of the studies included is small sample size with only RCTs included, which could lead to selection bias. Publication bias is not analyzed before.

In the current study, we performed a systematic review and meta-analysis to compare the complication of DHS and CS for femoral neck fracture. This study aimed to provide an evidence for treatment of femoral neck fractures for surgeon.

## Materials and methods

### Eligibility criteria and literature search

According to the Preferred Reporting Items for Systematic Reviews and Meta-Analyses statement (PRISMA) checklist and flow diagram [12], studies that compared DHS and CS for femoral neck fractures were searched in online databases such as Cochrane Central Register of Controlled Trials, Pubmed, and Ovid. Studies published from initial to Jan 7, 2020 were enrolled for inclusion. The following medical subject heading (Mesh) was used for searching “femoral neck fractures,” “femur,” “hip,” “dynamic,” “sliding,” “cannulated,” “cancellous,” and “intracapsular.” The search was limited to language of English. A hand search was also performed to avoid missing additional relevant trials by screening the reference lists of all the selected articles.

The inclusion criteria for this meta-analysis were (1) patients: adult patients diagnosed with femoral neck fracture; (2) intervention: patients treated by DHS (or CS); (3) comparison treatment: patients treated by CS (or DHS); (4) outcomes: mortality, non-union, AVN, and revision; 5) study design: RCTs, prospective, and retrospective were all included; the exclusion criteria were (1) duplicate publications, meta-analysis, systematic reviews, and case reports; (2) studies for whom full text was not available; (3) pathological fractures; and (4) studies presenting data that were incomplete and/or could not be extracted were excluded.

### Outcomes of interest, data extraction, and quality assessment

The interest outcome for this meta-analysis was mortality, non-union, AVN, and revision rate. Two reviewers independently screened the titles and abstracts according to the selection criteria, and any disagreements were discussed. The study design, country, age, gender, type of implant, follow-up, and outcomes were independently extracted by two reviewers. The quality of included RCTs was assessed by Cochrane Collaboration recommendations [13], and retrospective studies were assessed by Newcastle-Ottawa (NOS) [14]. For RCTs, random sequence generation (selection bias), allocation concealment (selection bias), blinding of outcome assessment (detection bias), incomplete outcome data (attrition bias), selective reporting (reporting bias), and other sources of bias were assessed. For retrospective studies, a total of nine scores including the selection, comparability, and outcome were assessed. When the score was greater than 7 points, the quality of the retrospective studies was considered high.

### Statistical analysis

All data were analyzed using the Review Manager software (Version 5.2, The Nordic Cochrane Centre, The Cochrane Collaboration, 2012). The odd ratio (OR) and 95% CI were calculated for dichotomous outcomes. Fixed-effect models and random-effect models were used when *I*^2^ *<* 50% and *I*^2^ *>* 50%, respectively. And when *I*^2^ *>* 50%, the sensitivity analysis or subgroup was conducted. Publication bias was also conducted by Review Manager (version 5.2). A *P* value of < .05 was considered statistically significant.

## Results

### Characteristics and qualities of the studies included

**Fig. 1:**
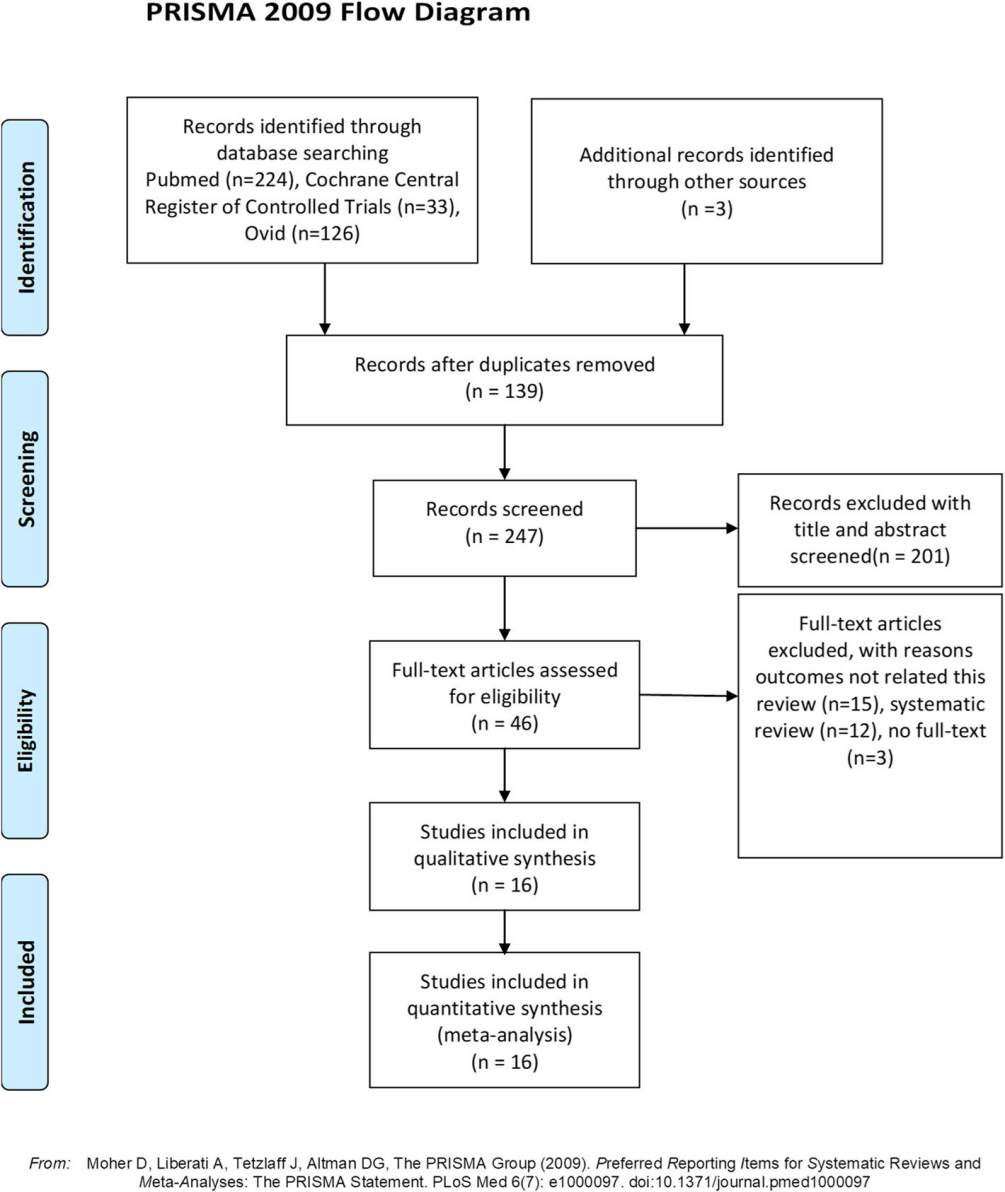
Flow diagram of the selection process of the studies included for the meta-analysis

**Fig. 2:**
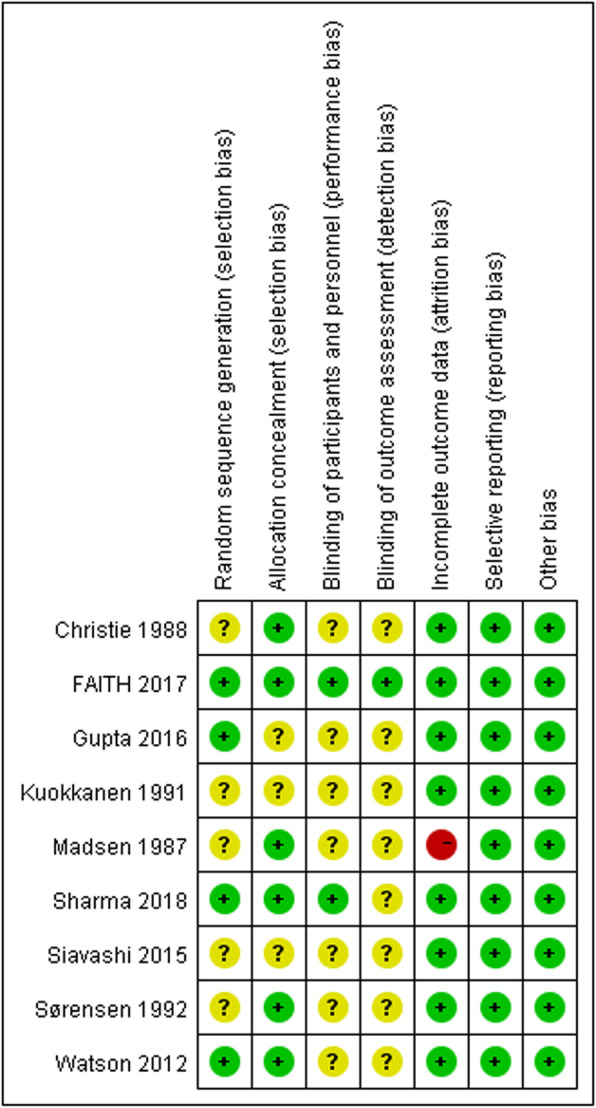
Quality assessment of risk of bias in the studies included

A total of 319 studies were retrieved, and 240 studies remained after duplication. After screening abstract full text, sixteen studies (nine RCTs and seven retrospective cohort studies) were included for meta-analysis, which was shown in Fig. 1. There were 2657 hips analyzed in the sixteen studies: 1337 hips for DHS and 1320 hips for CS. The full text of sixteen studies was available, and the basic characteristics of included studies were shown in Table 1. There was a moderate risk bias for the RCTs: four studies mentioned random sequence regeneration [17, 18, 23, 27], six studies mentioned allocation concealment [17, 18, 23–26], two studies used the blinding of participants [17, 18], one study used observed blinding [17], eight studies reported complete outcome [17, 18, 20, 21, 23, 24, 26, 27], and nine studies reported all outcome date without reporting bias [17, 18, 20, 21, 23–27]. For retrospective cohort studies, three studies had 9 scores [9, 10, 28], two studies had 8 scores [15, 19], and two studies had 7 scores [16, 22]. The details were shown in Fig. 2 and Table 2.

**Table 1:** Characteristics of the studies included

Included study	Country	Type	Mean age (year)	Hips analyzed(DHS/CS)	gender(Male/female)	Interventions	Follow-up	Outcome
Lee 2007 [9]	Taiwan	Retrospective	72.8/70.6	40/44	35/49	DHS *vs* multiple cannulated screws	12	①②③④
Widhalm 2019 [10]	UK	Retrospective	76.9(18-100)	254/199	110/343	DHS *vs* double cannulated screw	24	①②③④
Watson 2012 [15]	Australia	RCT	77.9(53-89)/76.7(53-93)	31/29	11/49	DHS *vs* Cancellous screws	24	①②③④
Sharma 2018 [16]	India	RCT	NA(21-57)	27/29	-	DHS *vs* Cancellous screws	24	②③
FAITH 2017 [17]	Canada	RCT	72.2±12.0/72±12.3	542/537	422/648	SHS *vs* Cancellous screws	24	①②③④
Gupta 2016 [18]	India	RCT	40.7(16-60)/39.3(16-60)	40/45	55/30	SHS *vs* multiple cancellous screws	12 to 36	①②③④
Sørensen 1992 [19]	Denmark	RCT	77(52-94)	35/38	18/55	SHS *vs* three Gouffon screws	36	①②③④
Madsen 1987 [20]	Denmark	RCT	75(25-91)/74(34-92)	51/52	25/78	SHS *vs* Cancellous screws	24	②④
Christie 1988 [21]	Scotland	RCT	69(26-80)	61/66	-	SHS *vs* double pin	33	②④
Siavashi 2015 [22]	Iran	RCT	30(18-59)/28(18-60)	30/28	46/12	DHS *vs* Cancellous screws	12 to 36	③④
Kuokkanen 1991 [23]	Finland	RCT	60/72.5	17/16	-	SHS *vs* three cancellous screws	24	④
Chen 2017 [24]	China	Retrospective	58.3±9.3 /56.8±8.6	42/44	38/48	DHS *vs* Cannulated compression screw	24 to 36	②③
Hoshino 2016 [25]	USA	Retrospective	40±12/39±11	48/15	41/21	DHS *vs* Pauwel screws	4 to 66	②③④
Bisaccia 2018 [26]	Italy	Retrospective	67.8(45-80)	42/75	40/77	DHS *vs* cannulated screws	12	②③④
Stiasny 2008 [27]	Denmark	Retrospective	NA(30-95)	44/70	41/71	DHS *vs* 3 AO screw	21.5	④
Tolga 2012 [28]	Turkey	Retrospective	46(25-67)/45(18-68)	33/33	35/31	DHS *vs* Cancellous screws	33.6	②③④

*SHS* sliding hip screws, *DHS* dynamic hip screws, *RCT* randomized clinical trial; ①Mortality②Non-union③AVN④Revision

**Table 2:** Risk of bias assessment using the Newcastle–Ottawa Scale for cohort studies included in a meta-analysis

Study	Selection				Comparability		Outcome			Total score
	Exposed cohort	Non-exposed cohort	Ascertainment of exposure	Outcome of interest	The most important factor	Additional factor	Assessment of outcome	Length of follow up	Adequacy of follow up	
Lee 2007 [9]	★	★	★	★	★	★	★	★	★	9
Widhalm 2019 [10]	★	★	★	★	★	★	★	★	★	9
Chen 2017 [24]	★	★	★	★	★	★	★	★	★	9
Hoshino 2016 [25]	★	★	★	★	★	★	★		★	8
Bisaccia 2018 [26]	★	★	★	★	★	★	★	★		8
Stiasny 2008 [27]	★	★	★	★	★		★		★	7
Tolga 2012 [28]	★	★	★	★	★		★		★	7

### Mortality

**Fig. 3:**
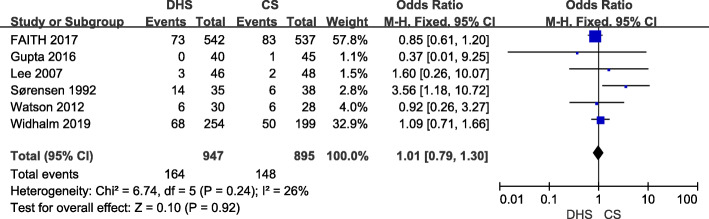
Forest plot of odds ratio with confidence intervals for mortality

Mortality at the last follow-up was reported in six studies [9, 10, 17, 23, 26, 27] including 1842 hips (947 for DHS and 895 for CS). The follow-up period of included studies varied from 12 to 36 months. With low heterogeneity (*p* = 0.24, *I*^2^ = 26%), fixed-effect model was conducted. The mortality rate of DHS and CS methods was 17.3% and 16.5%, respectively, but there was no significant difference between these two methods (Fig. 3).

### Non-union

**Fig. 4:**
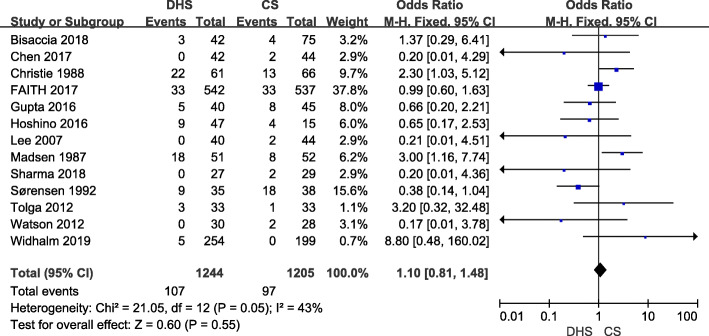
Forest plot of odds ratio with confidence intervals for non-union

Thirteen studies [9, 10, 15, 17–19, 22–28] enrolled 2450 hips (1245 for DHS and 1205 for CS) which reported the non-union rate. The follow-up period of included studies varied from 4 to 66 months. There results indicated no significant differences between the DHS and CS treatment (OR 1.10; 95% CI 0.81–1.48; *p* = 0.55). Lower heterogeneity was found (*I*^2^ = 43%; *p* = 0.05), and fixed-effect model was conducted (Fig. 4).

### AVN

**Fig. 5:**
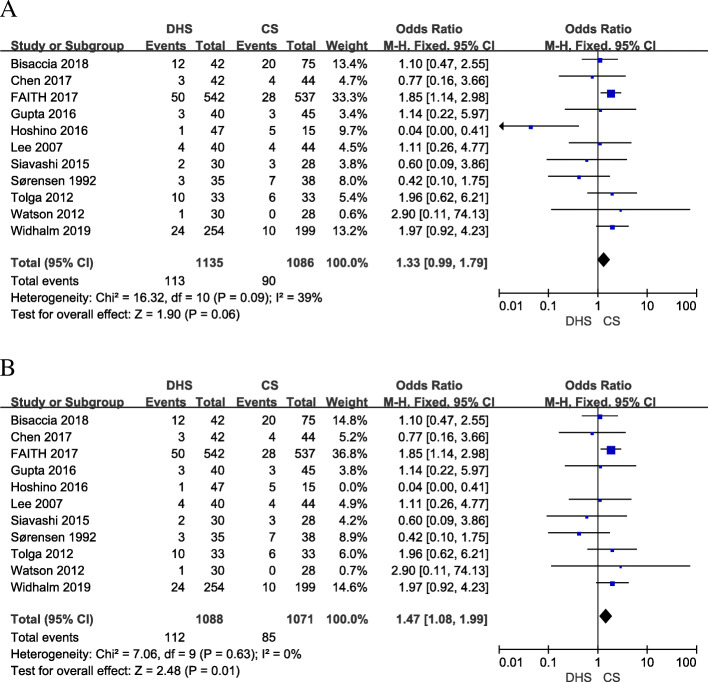
Forest plot of odds ratio with confidence intervals for AVN. **a** meta-analysis of AVN. **b** sensitive analysis of AVN

Eleven studies [9, 10, 15, 17, 19, 21–23, 26–28] included AVN rate. The follow-up period of included studies varied from 4 to 66 months. There was a little heterogeneity across the included 11 studies (*I*^2^ = 39%; *p* = 0.09). Compared with CS, the DHS has no benefit on the AVN (OR 1.33; 95% CI 0.99–1.79; *p* = 0.06) (Fig. 5a). In our meta-analysis, we found that a study by Hoshino [19] is different from other included studies. The hips included in DHS treatment are more than three times to CS treatment, and only 15 hips were analyzed in the CS group, which would lead to selection bias. After this study removed, a sensitive meta-analysis was conducted. The result showed the heterogeneity decreased from 39 to 0%, and the CS showed less AVN with significant difference compared with DHS treatment (OR 1.47; 95% CI 1.08–1.99; *p* = 0.01, *I*^2^ = 0%) (Fig. 5b).

### Revision rate and publication bias

**Fig. 6:**
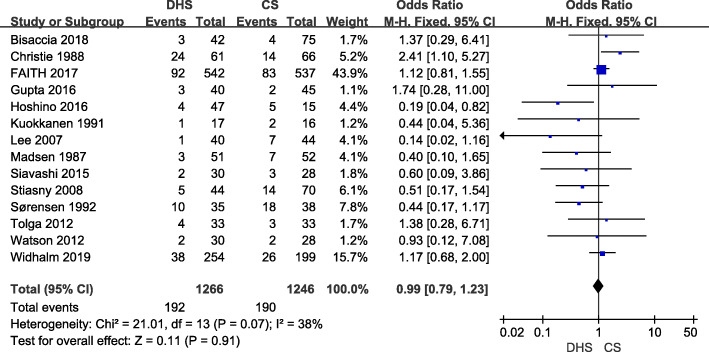
Forest plot of odds ratio with confidence intervals for revision

**Fig. 7:**
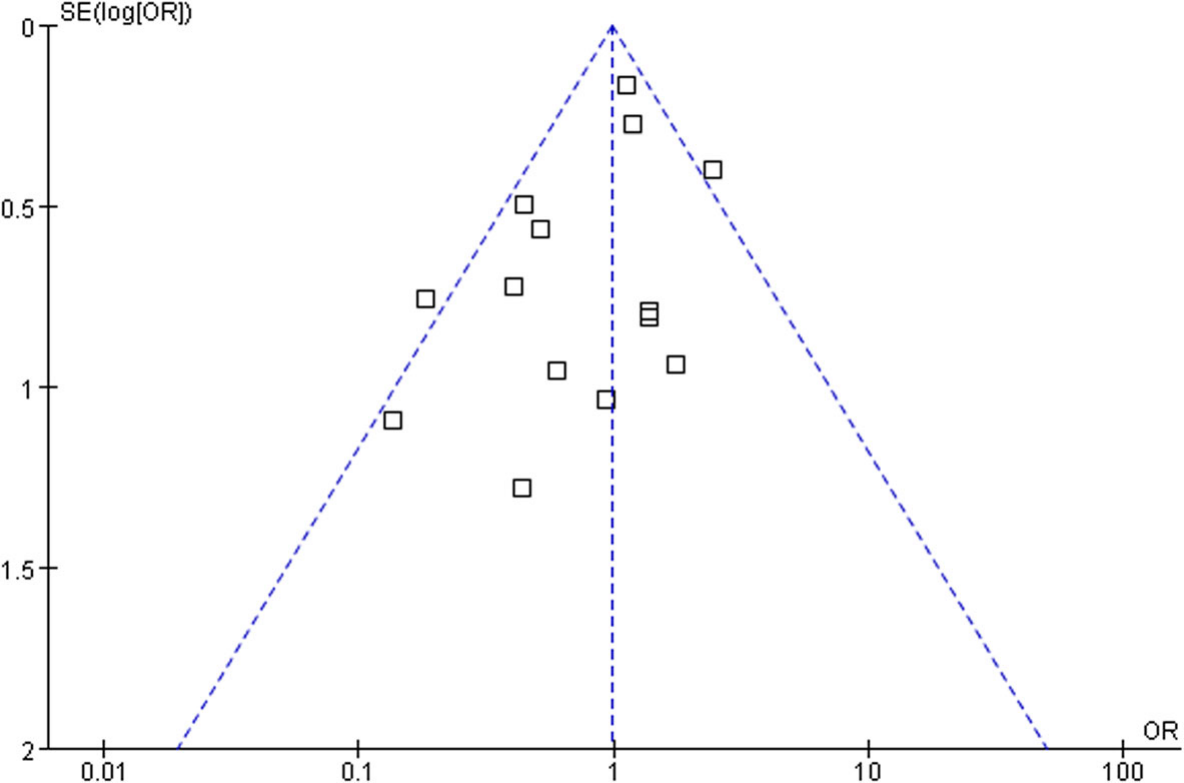
Publication bias of mortality

Fourteen studies [9, 10, 15–17, 19–27] including 2513 hips compared the revision rate between DHS and CS treatments. The follow-up period of included studies varied from 4 to 66 months. The revision rate of DHS and CS was 15.15% and 15.25%, respectively. There was no significant difference between DHS and CS (OR 0.99; 95% CI 0.79–1.23; *p* = 0.91) when a fixed-model was used (*I*^2^ = 39%) (Fig. 6). Revision rate enrolled most of included sixteen studies, so publication was performed for revision rate. We found that the triangle is basically symmetrical, and no obvious publication bias existed (Fig. 7).

## Discussion

Due to the anatomical and blood supply characteristics of femoral neck and femoral head, the incidence of complications and disability after femoral neck fracture surgery are relatively high [29, 30]. Internal fixation is less invasive and cheaper, could also protect femoral head, and delay the need for future arthroplasty, so DHS or CS is commonly used for femoral neck fractures in young patients and some elderly patients [31]. However, there is no consensus on the chosen between DHS and CS. In this study, we performed a meta-analysis to compare the complications between DHS and CS for the treatment of femoral neck fractures. Our results revealed that CS is superior to DHS on AVN rate, but has similar performance on mortality, non-union, and revision rate when compared with DHS.

Compared with previous systematic reviews, our study has some advantages. First, some latest studies [10, 15, 18] were not enrolled for analysis in previous study. Compared with previous studies [8, 11, 32], our study has a larger sample size, and sixteen trials in total were enrolled, which provided more strong evidence for surgeon. Second, the current study had RCTs and retrospective trials with a longer follow-up duration of 4–66 months. Third, the complications were divided into mortality, re-invention or revision, non-union, and AVN, which provided more guidance for surgeons involved in these procedures unlike that in the study by Zhang et al. [8]. In Zhang et al.’s study, AVN and non-union were combined and collectively referred to as complications. However, AVN and non-union are obviously different and critical adverse events, and a subgroup and sensitive analysis is necessary. In our sensitive analysis, we found that the incidence of AVN rate is lower in CS treatments when compared with those in DHS treatment.

Our findings were basically consistent with current trends. For mortality, previous studies have reported that the mortality rate of DHS/CS ranged from 0 to 40% [26, 27], and no significant difference between those two treatment. We considered that the reason for the larger range may be due to the difference in sample size and follow-up time. In our study, the mortality rate of DHS and CS was 17% and 16%, respectively, which was consistent with previous conclusions [17]. For non-union, we found DHS and CS have the same non-union rate (8.6% and 8%, respectively), which was similar to previous studies [27, 28, 33]. However, we also have some different findings. For the AVN, we found that the incidence of AVN in the CS group (8.3%) was lower than that in the DHS group (9.9%), although there was no statistical difference between them in previous RCTs or meta-analysis [8, 23, 27]. In our meta-analysis, a study by Hoshino et al. [19] reported that AVN rate for CS was 33%, in which the incidence was much higher than that reported by other studies, which may be related to the different surgeon. We conducted sensitive analysis by excluded this study, and the AVN rate in the CS group was significantly lower than that in the DHS group with heterogeneity decreased from 39 to 0%. We consider that the main reason for this is the advantage of expanded sample size, and two large sample studies support our point of view [10, 17]. We considered that the lower AVN rate in CS group was related to less invasive, which protected the blood supply as much as possible [6, 8]. In addition, the incidence rate of AVN would be significantly different and depend on displacement of the femoral neck at the time of the injury, and the AVN in displaced fracture was obvious higher than that in the non-displaced fracture [15]. For another outcome, revision was defined as any reason that required internal fixation, hemiarthroplasty, or total hip replacement (THA). Previous studies used reoperation rate for analysis; however, reoperation includes various reasons such as infection and other factors requiring reoperation. Our study used revision rate for meta-analysis, which is helpful to compare the incidence of internal fixation and hemiarthroplasty/THA between the two surgical methods in more detail. Mohamed et al. [11] reported that the reoperation rate of DHS and CS is equivalent, while Zhang et al. [8] thought that the reoperation rate of DHS is lower than CS. However, we found that a study [34] included by Zhang et al. needed careful consideration because Targon Femoral Neck and DHS are obviously different.

Limitations were also existed in our study. First, non-English language studies as well as studies that could not obtain full text were not included in this meta-analysis, which could lead to selection bias. Second, previous study reported DHS and CS were also different for displaced and non-displaced fractures. The DHS has slight advantages for the management for displaced femoral neck fractures. However, due to the lack of detailed information of included study, we could not make subgroup analysis to compare the difference between the two treatment methods for displaced/non-displaced fractures. Third, the treatment methods of CS group were not completely consistent, some are two cannulated screws and some are three cannulated screws, which might have unpredictable bias. Forth, the age and fracture type could affect the mortality and development of AVN, respectively. However, many studies included in this meta-analysis include all ages and different fracture types (displaced and non-displaced hips). In a word, further analysis is required to provide stronger evidence for clinical treatment.

## Conclusion

DHS and CS have similar complication including mortality, revision rate, and non-union, but CS has superior to DHS on ANV. In future, more and more study needed to provide strong evidence because of some limitation existed in this study.

## Data Availability

All data are fully available without restriction.

## Abbreviations

DHS: Dynamic hip screw
CS: Cannulated screws
AVN: Avascular necrosis
RCTs: Randomized controlled trials
CI: Confidence interval
OR: Odds ratio

## References

[1] Singer BR, McLauchlan GJ, Robinson CM, Christie J (1998). Epidemiology of fractures in 15,000 adults: the influence of age and gender. J Bone Jnt Surg Brit Vol.

[2] Cummings SR, Nevitt MC, Browner WS, Stone K, Fox KM, Ensrud KE (1995). Risk factors for hip fracture in white women. Study of Osteoporotic Fractures Research Group. N Engl J Med.

[3] Dickson JA (1953). The unsolved fracture; a protest against defeatism. J Bone Joint Surg Am.

[4] Jansen H, Frey SP, Meffert RH (2010). Subtrochanteric fracture: a rare but severe complication after screw fixation of femoral neck fractures in the elderly. Acta Orthop Belg.

[5] Dedrick DK, Mackenzie JR, Burney RE (1986). Complications of femoral neck fracture in young adults. J Trauma.

[6] Tai TW, Lien FC, Lee PY, Jou IM, Lin CJ, Huang YH (2010). Using a cannulated screw as a drill guide and sleeve: a simple technique for multiple-screw fixation for intracapsular femoral neck fracture. Orthopedics..

[7] Brandt E, Verdonschot N (2011). Biomechanical analysis of the sliding hip screw, cannulated screws and Targon1 FN in intracapsular hip fractures in cadaver femora. Injury..

[8] Zhang LL, Zhang Y, Ma X, Liu Y (2017). Multiple cannulated screws vs. dynamic hip screws for femoral neck fractures : A meta-analysis. Der Orthopade.

[9] Yih-Shiunn L, Chien-Rae H, Wen-Yun L (2007). Surgical treatment of undisplaced femoral neck fractures in the elderly. Int Orthop.

[10] Widhalm HK, Arnhold R, Beiglbock H, Munteanu A, Lang NW, Hajdu S (2019). A Comparison of Dynamic Hip Screw and Two Cannulated Screws in the Treatment of Undisplaced Intracapsular Neck Fractures-Two-Year Follow-Up of 453 Patients. J Clin Med.

[11] Shehata MSA, Aboelnas MM, Abdulkarim AN, Abdallah AR, Ahmed H, Holton J (2019). Sliding hip screws versus cancellous screws for femoral neck fractures: a systematic review and meta-analysis. Eur J Orthop Surg Traumatol.

[12] Moher D, Liberati A, Tetzlaff J, Altman DG, Group P (2010). Preferred reporting items for systematic reviews and meta-analyses: the PRISMA statement. Int J Surg (London, England).

[13] Furlan AD, Malmivaara A, Chou R, Maher CG, Deyo RA, Schoene M (2015). 2015 Updated Method Guideline for Systematic Reviews in the Cochrane Back and Neck Group. Spine..

[14] Stang A (2010). Critical evaluation of the Newcastle-Ottawa scale for the assessment of the quality of nonrandomized studies in meta-analyses. Eur J Epidemiol.

[15] Bisaccia M, Ceccarini P, Rinonapoli G, Di Giacomo LM, Teodori J, Schiavone A (2018). Dhs plus anti-rotational screw vs cannulated screws for femoral neck fractures: an analysis of clinical outcome and incidence regarding avn. Acta Orthop Belg.

[16] Stiasny J, Dragan S, Kulej M, Martynkiewicz J, Plochowski J, Dragan SL (2008). Comparison analysis of the operative treatment results of the femoral neck fractures using side-plate and compression screw and cannulated AO screws. Ortopedia Traumatol Rehabil.

[17] Investigators FuAIftToHfF (2017). Fracture fixation in the operative management of hip fractures (FAITH): an international, multicentre, randomised controlled trial. Lancet (London, England).

[18] Sharma A, Sethi A, Sharma S (2018). Comparative analysis of treatment of basicervical femur fractures in young adults with CCS, DHS, and PFN. Rev Bras Ortop.

[19] Hoshino CM, Christian MW, O'Toole RV, Manson TT (2016). Fixation of displaced femoral neck fractures in young adults: Fixed-angle devices or Pauwel screws?. Injury..

[20] Kuokkanen H, Korkala O, Antti-Poika I, Tolonen J, Lehtimaki MY, Silvennoinen T (1991). Three cancellous bone screws versus a screw-angle plate in the treatment of Garden I and II fractures of the femoral neck. Acta Orthop Belg.

[21] Siavashi B, Aalirezaei A, Moosavi M, Golbakhsh MR, Savadkoohi D, Zehtab MJ (2015). A comparative study between multiple cannulated screws and dynamic hip screw for fixation of femoral neck fracture in adults. Int Orthop.

[22] Kaplan T, Akesen B, Demirag B, Bilgen S, Durak K (2012). Comparative results of percutaneous cannulated screws, dynamic compression type plate and screw for the treatment of femoral neck fractures. Ulus Travma Acil Cerrahi Derg.

[23] Watson A, Zhang Y, Beattie S, Page RS (2013). Prospective randomized controlled trial comparing dynamic hip screw and screw fixation for undisplaced subcapital hip fractures. ANZ J Surg.

[24] Christie J, Howie CR, Armour PC (1988). Fixation of displaced subcapital femoral fractures. Compression screw fixation versus double divergent pins. J Bone Jnt Surg Brit Vol.

[25] Madsen F, Linde F, Andersen E, Birke H, Hvass I, Poulsen TD (1987). Fixation of displaced femoral neck fractures. A comparison between sliding screw plate and four cancellous bone screws. Acta Orthop Scand.

[26] Sorensen JL, Varmarken JE, Bomler J (1992). Internal fixation of femoral neck fractures. Dynamic Hip and Gouffon screws compared in 73 patients. Acta Orthop Scand.

[27] Gupta M, Arya RK, Kumar S, Jain VK, Sinha S, Naik AK (2016). Comparative study of multiple cancellous screws versus sliding hip screws in femoral neck fractures of young adults. Chin J Traumatol.

[28] Chen C, Yu L, Tang X, Liu MZ, Sun LZ, Liu C (2017). Dynamic hip system blade versus cannulated compression screw for the treatment of femoral neck fractures: A retrospective study. Acta Orthop Traumatol Turc.

[29] Giordano V, Giordano M, Aquino R, Grossi JO, Senna H, Koch HA (2019). How do Orthopedic Surgeons Manage Displaced Femoral Neck Fracture in the Middle-Aged Patient? Brazilian Survey of 78 Orthopaedic Surgeons. Rev Bras Ortop (Sao Paulo).

[30] Parker MJ (2009). Results of internal fixation of Pauwels type-3 vertical femoral neck fractures. J Bone Joint Surg Am.

[31] Zielinski SM, Meeuwis MA, Heetveld MJ, Verhofstad MH, Roukema GR, Patka P (2013). Adherence to a femoral neck fracture treatment guideline. Int Orthop.

[32] Li T, Zhang QS (2018). Is dynamic locking plate superior than other implants for intracapsular hip fracture: A meta-analysis. Medicine..

[33] Jettoo P, James P (2016). Dynamic hip screw fixation versus multiple screw fixation for intracapsular hip fracture. J Orthopaedic Surg (Hong Kong).

[34] Griffin XL, Parsons N, Achten J, Costa ML (2014). the Targon femoral neck hip screw versus cannulated screws for internal fixation of intracapsular fractures of the hip: a randomised controlled trial. Bone Jnt J.

